# Opposing regulatory functions of the TIM3 (HAVCR2) signalosome in primary effector T cells as revealed by quantitative interactomics

**DOI:** 10.1038/s41423-020-00575-7

**Published:** 2020-11-02

**Authors:** Yunhao Zhai, Javier Celis-Gutierrez, Guillaume Voisinne, Daiki Mori, Laura Girard, Odile Burlet-Schiltz, Anne Gonzalez de Peredo, Romain Roncagalli, Bernard Malissen

**Affiliations:** 1 Centre d’Immunologie de Marseille-Luminy, Aix Marseille Université, INSERM, CNRS, 13288 Marseille, France; 2 Centre d’Immunophénomique, Aix Marseille Université, INSERM, CNRS, Marseille, France; 3 Institut de Pharmacologie et de Biologie Structurale, Département Biologie Structurale Biophysique, Protéomique Génopole Toulouse Midi Pyrénées CNRS UMR 5089, Toulouse, France

**Keywords:** Signal transduction, Adaptive immunity

Deciphering how T-cell antigen receptor signals are modulated by coinhibitors is a fundamental goal in immunology and of considerable clinical interest because blocking coinhibitory signals via therapeutic antibodies have become a standard cancer immunotherapeutic strategy. Most of the attention devoted to T-cell immunoglobulin and mucin domain-3 (TIM3; also known as HAVCR2 or CD366) molecules stems from their expression on exhausted T cells in settings of chronic viral infection and tumors. Moreover, T cells expressing high levels of both PD-1 and TIM3 coinhibitors appear more dysfunctional than those expressing PD-1 alone. Combination therapies intending to block both PD-1 and TIM3 are thus actively being explored in the cancer treatment setting. Upon interaction with Galectin-9 (GAL-9) and carcinoembryonic antigen-related cell adhesion molecule 1 (CEACAM-1), the tyrosines found in the TIM3 cytoplasmic tail are phosphorylated.^1^ Because these conserved tyrosines do not form a recognizable inhibitory signaling motif, the mechanism by which TIM3 transmits inhibitory signals has not been elucidated. Paradoxically, TIM3 also has costimulatory activity in T cells.^2,3^ Published biochemical studies attempting to unveil the mode of action of TIM3 have relied on approaches addressing one candidate effector at a time with limited quantitative insight, and most used transformed cells. Using mice expressing an affinity Twin-Strep-tag (OST) at the TIM3-protein C-terminus (TIM3^OST^ mice) (Figs. 1a and S1a) and affinity purification coupled with mass spectrometry (AP-MS), we herein defined the composition and dynamics of the signaling protein complex (signalosome) used by TIM3 in primary effector T cells. These results provide a more complete model of TIM3 signaling and explain its paradoxical coinhibitory and costimulatory functions.

**Fig. 1:**
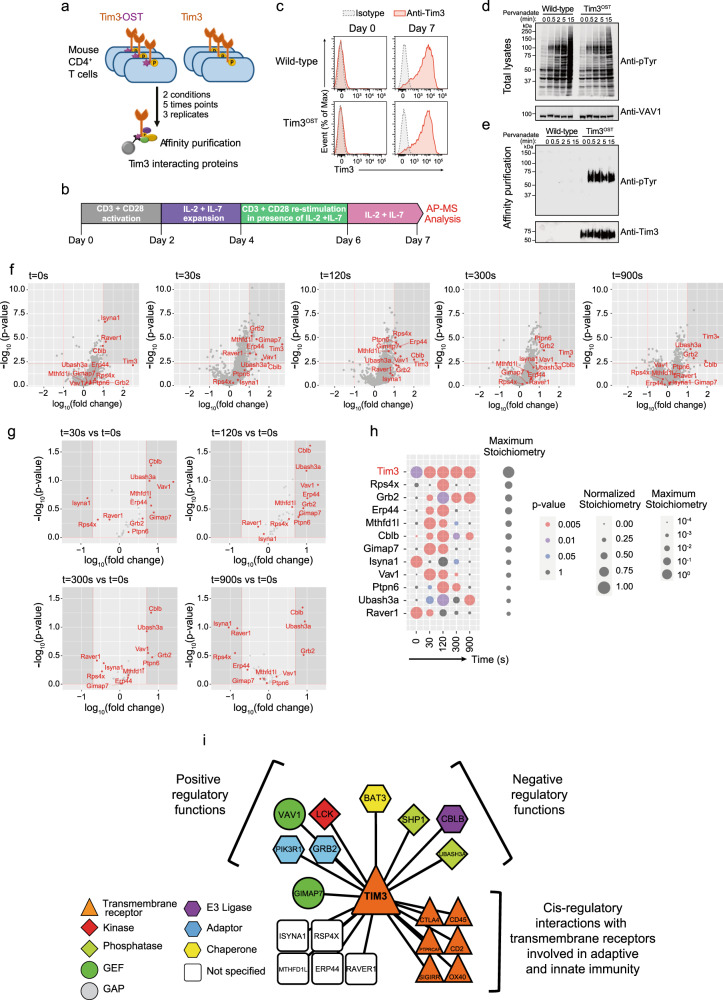
Composition and dynamics of the TIM3 signalosome of CD4^+^ T cells. **a** Overview of AP-MS analysis of CD4^+^ effector T cells expressing TIM3^OST^ or TIM3 molecules. **b** Workflow used to obtain TIM3^+^ CD4^+^ effector T cells in numbers required for AP-MS. **c** CD4^+^ T cells purified from WT and TIM3^OST^ mice were left unstimulated (Day 0) or expanded as described in **b** (Day 7) and analyzed by flow cytometry for TIM3 expression. Gray shaded curves, isotype-matched control antibody. **d** Immunoblot analysis of equal amounts of proteins from total lysates of TIM3^+^ CD4^+^ effector T cells harvested from WT and TIM3^OST^ mice left unstimulated (0 s) or stimulated with pervanadate as specified and probed with an antibody against phosphorylated tyrosine (Anti-p-Tyr) or anti-VAV1 (loading control). Left, molecular size in kilodaltons (kDa). **e** Immunoblot analysis of equal amounts of proteins from total lysates of cells as described in **d**; the proteins were subjected to affinity purification and probed with an antibody against phosphorylated tyrosine (Anti-p-Tyr) or anti-TIM3 (affinity purification control). The data in **c**–**e** are representative of three independent experiments. **f** Volcano plots showing proteins significantly enriched after affinity purification in CD4^+^ T cells expressing TIM3^OST^ molecules compared to those in affinity-purified control CD4^+^ T cells expressing similar levels of untagged TIM3 proteins prior to (0 s) and after pervanadate stimulation. Thirty-seven high-confidence TIM3 prey proteins were identified (highlighted in red). **g** Volcano plots identifying the 11 high-confidence prey proteins that interacted with TIM3 in an activation-regulated manner (highlighted in red). In **f** and **g**, the *x* axis shows the average fold change (in log_10_ scale) in protein intensity (**f**) and protein stoichiometry (**g**), whereas the *y* axis shows statistical significance. **h** Dot plot showing the interaction stoichiometries of TIM3 (denoted in red) with its 11 activation-regulated prey proteins over the course of activation (denoted in black and ranked according to the maximum stoichiometry; see key for maximum stoichiometries and *p* value color codes). The interaction stoichiometries were row-normalized to the maximum values observed over the course of pervanadate stimulation (see key for normalized stoichiometry). **i** TIM3-protein interaction network of mouse CD4^+^ effector T cells. Key indicates classification according to function. The roles of the eRPS4X, ERP44, MTHFD1L, GIMAP7, ISNYA1, and RAVER1 prey proteins found in the TIM3 signalosome remain to be elucidated

Normal numbers of T cells were present in TIM3^OST^ mice, and they proliferated appropriately in response to TCR stimulation (Fig. S1b, c). Following a brief in vitro expansion and restimulation with anti-CD3 and anti-CD28 antibodies, CD4^+^ T cells from TIM3^OST^ mice expressed levels of TIM3 comparable to those in their wild-type (WT) counterparts (Figs. 1b, c and S1d). Consistent with the presence of extensive N-glycosylation,^4^ treatment of affinity-purified TIM3-OST molecules with glycopeptidase F reduced their molecular weight (Fig. S1e). Therefore, CD4^+^ effector T cells from TIM3^OST^ mice expressed proper TIM3 molecules. Considering that the interaction of TIM3 with GAL-9 and CEACAM-1 remains controversial,^5^ the effector CD4^+^ T cells subjected to AP-MS were activated with pervanadate to trigger the phosphorylation of TIM3 tyrosines.^1^ After activation with pervanadate, TIM3^OST^ and WT CD4^+^ effector T cells showed similar patterns of inducible tyrosine phosphorylation (Fig. 1d). As expected, TIM3-OST molecules were affinity-purified from TIM3^OST^ samples and showed robust levels of tyrosine phosphorylation after pervanadate stimulation (Fig. 1e). To identify and quantify the proteins (“the prey”) associated with TIM3-OST molecules (“the bait”) over the course of pervanadate stimulation, CD4^+^ effector T cells that expressed TIM3-OST molecules were left untreated or treated for 30, 120, 300, and 900 s with pervanadate (Fig. 1f). To distinguish true TIM3-interacting proteins from nonspecific contaminants, we compared our data with those of control AP-MS experiments involving WT CD4^+^ T cells that were subjected to the same expansion-restimulation protocol as TIM3^OST^ CD4^+^ T cells and expressed comparable levels of untagged TIM3 molecules (Fig. 1c). For each time point, three independent biological replicates were performed, and the proteins copurifying with TIM3^OST^ were analyzed by MS.^6^ We identified 37 high-confidence TIM3 prey proteins that showed a greater than tenfold enrichment with a *p* value below 0.005 in at least one of the five conditions of stimulation (Fig. 1f and Supplementary dataset 1). Among them, 11 prey proteins (RPS4X, GRB2, ERP44, MTHFD, CBLB, GIMAP7, ISYNA1, VAV1, SHP1 (also known as PTPN6), UBASH3A (also known as STS-2), and RAVER1 showed interaction stoichiometries that changed at least fivefold following pervanadate stimulation (Fig. 1g). Importantly, all 11 activation-regulated TIM3 prey proteins corresponded to novel TIM3 interactors. Analysis of interaction stoichiometries over the course of stimulation further showed that most of the activation-inducible prey proteins bound to TIM3 after 30–120 s of activation (Fig. 1h).

We had to slightly relax our stringent cutoff values to identify previously reported TIM3 interactors.^1,7^ All these known interactors constitutively bound to TIM3 and corresponded to the p85α subunit of phosphoinositide 3-kinase (P85A; 10-fold enrichment, *p* value: 2.5 × 10^−^^7^), the chaperone BAT3 (BAG6; 8.7-fold enrichment, *p* value: 9.0 × 10^−7^), and the protein tyrosine kinase LCK (8.5-fold enrichment, *p* value: 3.0 × 10^−6^) (Fig. 1i and S2; Supplementary dataset 1). Finally, we confirmed that TIM3 constitutively associated in cis with the receptor PTPase CD45 (also known as PTPRC),^8^ and we extended this finding to several other transmembrane glycoproteins expressed at the T-cell surface, including PTPRCAP (which associates with CD45), TNFRSF4 (also known as OX40), the coinhibitor CTLA-4, the cell adhesion protein CD2, and SIGIRR (Fig. 1i). This unexpected finding suggests that TIM3 modulates the functions of those receptors that are involved in adaptive and innate immunity. Among them, SIGIRR is also known as IL-1R8 or TIR8 and negatively regulates Toll-like, IL-1 and IL-18 receptor signaling.^9^ The presence of SIGIRR-TIM3 complexes on effector T cells points to the intriguing possibility that they play a broader negative regulatory role than SIGIRR or TIM3 alone.

In conclusion, our systems-level study provides a comprehensive analysis of the composition of the TIM3 signalosome of primary effector CD4^+^ T cells. The detection of the E3 ubiquitin ligase CBLB and of the protein tyrosine phosphatases SHP1 and UBASH3A among TIM3 prey proteins readily explains the coinhibitory function of TIM3. Conversely, the presence of the VAV1, LCK, and P85A interactors likely accounts for the costimulatory role of TIM3, including the activation of AKT/mTOR signaling.^1^ TIM3 might thus assemble a signalosome in effector T cells that has opposing regulatory functions. Alternatively, TIM3 signalosome isoforms endowed with negative or positive regulatory roles might coexist in effector T cells. The molecular foundation provided herein for TIM3 having both inhibitory and stimulatory roles provides clues for future functional experiments and the study of TIM3 functions in settings such as those that lead to immune exhaustion.

## Supplementary information

Supplemental Material

Dataset 1
