# Supplementary material for: Opposing regulatory functions of the TIM3 (HAVCR2) signalosome in primary effector T cells as revealed by quantitative interactomics
Source: Cell Mol Immunol. 2020 Nov 2;18(6):1581–3. doi: 10.1038/s41423-020-00575-7 (PMC8167182; doi:10.1038/s41423-020-00575-7)
Supplement: Supplementary file 1 — Supplemental Material [file 41423_2020_575_MOESM1_ESM.pdf]

Opposite regulatory functions of the TIM3 (HAVCR2) signalosome revealed by quantitative interactomics in primary effector T cells

Yunhao Zhai[1,4], Javier Celis-Gutierrez[1,2,4], Guillaume Voisinne[1], Daiki Mori[1,2], Laura Girard[1,2], Odile Burlet-Schiltz[3], Anne Gonzalez de Peredo[3], Romain Roncagalli,[1*] and Bernard Malissen[1,2,*]

[1]Centre d'Immunologie de Marseille-Luminy, Aix Marseille Université, INSERM, CNRS, 13288 Marseille, France

[2]Centre d'Immunophénomique, Aix Marseille Université, INSERM, CNRS UMR, 13288 Marseille, France

[3]Institut de Pharmacologie et de Biologie Structurale, Département Biologie Structurale Biophysique, Protéomique Génopole Toulouse Midi Pyrénées CNRS UMR 5089, 205 Route de Narbonne, 31077 Toulouse Cedex, France

[4]These authors contributed equally

[*]Correspondence to B.M. (bernardm@ciml.univ-mrs.fr) or R.R. (roncagalli@ciml.univ-mrs.fr)

SUPPLEMENTARY MATERIALS AND METHODS

Mice and ethical statement

Mice were maintained on a C57BL/6 background. Generation of the TIM3^{OST} (B6-*Havcr2*^{tm2Ciphe}) mice is described below. Mice were handled under specific pathogen-free conditions in accordance with national and European laws for laboratory animal welfare and

experimentation (EEC Council Directive 2010/63/EU, September 2010), and the protocols were approved by the Marseille Ethical Committee for Animal Experimentation.

Havcr2$^{\mathbf{OST}}$ targeting vectors

A double-stranded DNA repair template (targeting vector) was assembled. It consists of a Twin-Strep-tag coding sequence (OST)[1] inserted at the 3' end of the last exon (exon 7) of the *Havcr2* gene that codes for TIM3, and of 5' and 3' homology arms of 1100 and 900 bp, respectively. A self-excising ACN cassette[2] was introduced at the beginning of the 3′ UTR sequence, and the final targeting vector abutted to a cassette coding for the diphtheria toxin fragment A[3].

Isolation of recombinant embryonic stem (ES) cell clones

Two sgRNA-specifying oligonucleotide sequences (5'-CACCGCAGCCATCCTGACCGCCTC-3' and 5'-AAACGAGGCGGTCAGGATGGCTGC-3') were chosen using publicly available tool (http://crispor.tefor.net), and annealed to generate overhangs for ligation into the BbsI site of plasmid pX330 (pSpCas9; Addgene, plasmid ID 42230). JM8.F6 C57BL/6N ES cells[4] were electroporated with 20 µg of *Havcr2*^{OST} targeting vector and 2.5 µg of the pX330-sgRNA plasmid. After selection in G418 plus ganciclovir, ES cell clones were screened for proper homologous recombination by Southern blot and PCR analysis. A *neo*^{r} specific probe was used to ensure that adventitious non-homologous recombination events had not occurred in the selected ES clones.

Production of knock-in mice

Mutant ES cells were injected into BalbC/N blastocysts. Following germ-line transmission of the *Havcr2*^{OST} allele, screening for proper deletion of the ACN cassette and for the presence of the OST coding sequence was performed by PCR using the following pair of primers: sense 5'-TTATTACACTGGCCAACTTG-3' and antisense 5'-AAGTCAGAAATGAAGGCGAG-3'. This pair of primers amplified a 386 bp band and a 205 bp band in the case of the *Havcr2*^{OST}

and wild-type *Havcr* allele, respectively. The proper structure of the 3' end of the *Havcr2*^{OST} allele was confirmed via DNA sequencing.

Flow cytometry of mouse T and B cells

Stained cells from mouse spleen and lymph nodes were analyzed using an LSRII system (BD Biosciences). Data were analyzed with the Diva software (BD Biosciences). Cell viability was evaluated using SYTOX Blue (Life Technologies). The following antibodies were used: anti-CD3e (145-2C11) PE, anti-CD4 (RM4-5) BV650, anti-CD5 (53-7.3) Pe-Cy5, anti-CD8a (53-6.7) AF700, anti-CD19 (6D5) APC Fire750, anti-CD44 (IM7) PE-Cy7, anti-CD62L (MEL-14) FITC, anti-rat IgG2a, κ (R35-95) PE and anti-TIM3 (RMT3-23) PE from BD Biosciences, Biolegend and eBioscience.

Mouse CD4^{+} T cell proliferation

Purified CD4^{+} T cells were stimulated with plate-bound anti-CD3 (145-2C11, 3 μg/ml) and soluble anti-CD28 (37-51, 1 μg/ml) both from Exbio Praha. After 48 h of culture, T cell proliferation was assessed with CellTiter-Glo® Luminescent (Promega). The resulting luminescence, which is proportional to the ATP content of the culture, was measured with a Victor 2 luminometer (Wallac, Perkin Elmer Life Science).

Mouse CD4^{+} T cell isolation and short-term expansion prior to AP-MS analysis

CD4^{+} T cells were isolated from pooled lymph nodes and spleens with Dynabeads Untouched Mouse CD4^{+} T Cell Kits (Life Technologies) with a > 95% purity. Purified CD4^{+} T cells were activated with plate-bound anti-CD3 (145-2C11, 5 μg/ml) and soluble anti-CD28 (37-51, 1 μg/ml) antibodies under neutral Th0 conditions. After two days of culture, CD4^{+} T cells were grown for an additional 48h in the presence of IL-2 (5–10 U/ml) and IL-7 (0.5 ng/ml, PeproTech). CD4^{+} T cells were then restimulated for 48 h with plate-bound anti-CD3 (145-2C11, 1 μg/ml) and soluble anti-CD28 (37-51, 1 μg/ml) antibodies in presence of IL-2 (5–10 U/ml) and IL-7 (0.5 ng/ml). Cells were then harvested and rested overnight in the presence of IL-2 (5–10 U/ml) and IL-7 (0.5 ng/ml) prior to be used for AP-MS experiments.

Affinity purification of OST-tagged TIM3 protein complexes

Short-term expanded $CD4^+$ T cells (100 x 10^6) from $TIM3^{OST}$ and wild-type (WT) mice were kept at 37 °C for 5 min and either left unstimulated or stimulated with pervanadate for the specified times. Stimulation was stopped by the addition of a twice-concentrated lysis buffer (100 mM Tris, pH 7.5, 270 mM NaCl, 1 mM EDTA, 20% glycerol, 0.4% n-dodecyl-β-maltoside) supplemented with protease and phosphatase inhibitors. After 10 min of incubation on ice, cell lysates were centrifuged at 21,000 g for 5 min at 4 °C. Equal amounts of post-nuclear lysates were incubated with Strep-Tactin Sepharose beads (IBA GmbH) for 1.5 h at 4°C on a rotary wheel. Beads were then washed two times with 1 ml of lysis buffer with detergent and of protease and phosphatase inhibitors and three times with 1 ml of lysis buffer in the absence of detergent and of protease and phosphatase inhibitors. Proteins were eluted from the Strep-Tactin Sepharose beads with 2.5 mM D-biotin, a competitive ligand that binds to Strep-Tactin with a higher affinity than the OST sequence. Eluted samples and whole-cell lysates were loaded on 8% SDS–PAGE gel and subsequently analyzed by immunoblot with specific antibodies. The following antibodies were used for immunoblot analysis: anti-VAV1 (2502), anti-TIM3 (D3M9R) from Cell Signaling Technology and anti-Phosphotyrosine (4G10) from Millipore.

Sensitivity of TIM3 molecule to glycopeptidase F

To determine the sensitivity of TIM3 molecule to glycopeptidase F (PNGase F; EC 3.2.2.18, New England Biolabs), a glycoaminidase that cleaves the link between asparagine and N-acetylglucosamine, TIM3-OST molecules were affinity purified from $CD4^+$ T cells from $TIM3^{OST}$ mice using Sepharose beads coupled to Strep-Tactin. They were then treated with 1 unit of PNGase F, reduced, and loaded on 8% SDS–PAGE gel and subsequently analyzed by immunoblot with anti-TIM3 (D3M9R).

Tandem MS analysis

Following affinity purification, protein samples were processed for proteomic analysis as previously described[5]. In brief, samples were partially air dried, reconstituted in Laemmli buffer containing DTT (25 mM), and heated at 95°C for 5 min. Cysteines were alkylated for 30 min at room temperature by the addition of iodoacetamide (90 mM). Protein samples were shortly migrated on a SDS–PAGE gel, and in-gel-digested using 0.6 µg of modified sequencing-grade trypsin (Promega) in 50 mM ammonium bicarbonate overnight at 37°C. The resulting peptides were extracted from the gel and purified on a C18 ZipTip (Millipore). Tryptic peptides were resuspended in 20 µl of 2% acetonitrile and 0.05% trifluoroacetic acid and 5µL of each sample were analyzed using an UltiMate 3000 system (NCS-3500RS Nano/Cap System; Dionex) coupled to an Orbitrap Velos Pro mass spectrometer (Thermo Fisher Scientific). Peptides were loaded on a C18 precolumn (300 µm inner diameter × 5 mm, Dionex), and separated on an analytical C18 column (75 µm inner diameter × 50 cm, in-house packed with Reprosil C18) equilibrated in 95% solvent A (5% acetonitrile, 0.2% formic acid) and 5% solvent B (80% acetonitrile, 0.2% formic acid), using a 5–50% gradient of solvent B over 105 min at a flow rate of 300 nl/min. The LTQ Orbitrap Velos was operated in data-dependent acquisition mode; Survey scan MS was acquired in the Orbitrap on the 350–1,800 m/z range, with the resolution set to a value of 60,000. The 20 most intense ions survey scans were selected for fragmentation by collision-induced dissociation, and the resulting fragments were analyzed in the linear trap. Dynamic exclusion was used within 60 s to prevent repetitive selection of the same peptide. Duplicate LC-MS measurements were performed for each sample.

Filtering strategy for the identification of the TIM3 high-confidence interactome

Raw MS files were processed with MaxQuant software (version 1.5.2.8) for database search with the Andromeda search engine and quantitative analysis. Data were searched against Mus musculus entries of the UniProtKB protein database (Swiss-Prot+TrEMBL including

isoforms), plus the One-Strep-tag peptide sequence, and the set of common contaminants provided by MaxQuant. Carbamidomethylation of cysteines was set as a fixed modification, whereas oxidation of methionine, protein N-terminal acetylation, and phosphorylation of serine, threonine, and tyrosine were set as variable modifications. Specificity of trypsin digestion was set for cleavage after K or R, and two missed trypsin cleavage sites were allowed. The precursor mass tolerance was set to 20 ppm for the first search and 4.5 ppm for the main Andromeda database search. The mass tolerance in tandem MS mode was set to 0.5 Da. Minimum peptide length was set to 7 amino acids, and minimum number of unique or razor peptides was set to 1 for validation. The I=L option of MaxQuant was enabled to avoid erroneous assignation of undistinguishable peptides belonging to very homologous proteins. Andromeda results were validated by the target decoy approach using a reverse database, with a false discovery rate set at 1% at both PSM (peptide sequence match) and protein level. For label-free relative quantification of the samples, the match between runs option of MaxQuant was enabled with a match time window of 1 min, to allow cross-assignment of MS features detected in the different runs, after alignment of the runs with a time window of 20min. Protein quantification was based on unique and razor peptides. The minimum ratio count was set to 1 for LFQ calculation, and computation of the iBAQ metric was also enabled.

From the "proteinGroups.txt" files generated by MaxQuant with the options described above, proteins groups only identified by site were filtered as well as proteins identified from the reverse database and those identified as contaminant. When protein groups corresponded to the same gene name, protein intensities in a given sample were summed over the redundant protein groups. Protein intensities were normalized across all runs by the median intensity. Normalized intensities corresponding to different technical replicates were averaged (geometric mean) and missing values were replaced after estimating background binding from wild-type intensities. For the TIM3 bait and each condition of stimulation (time point), we used a two-

tailed Welch t-test to compare normalized log-transformed protein intensities detected in OST-tagged samples across all biological replicates to protein intensities measured in WT samples across all biological replicates and all conditions of stimulation. To avoid spurious identification of interactors due to missing value imputation, we repeated this process (missing value imputation followed by a two-tailed Welch t-test) 10 times and estimated fold-changes and p-values as their respective average (geometric mean) across all 10 tests. Specific interactors were identified as preys showing a greater than 10-fold enrichment with a p-value below 0.005 in at least one condition of stimulation.

Quantification and statistical analysis

In all experiments, data are presented as mean ± SEM unless stated otherwise. Statistical tests were selected based on appropriate assumptions with respect to data distribution and variance characteristics. The number of biological replicates and of mice is indicated in the figure legends. The statistical analysis used for the identification of the TIM3 high-confidence interactors is described in the paragraph ‘Filtering strategy for the identification of the TIM3 high-confidence interactome’.

Data availability

The mass spectrometry proteomics data have been deposited to the ProteomeXchange Consortium via the PRIDE partner repository (http://www.ebi.ac.uk/pride) with the dataset identifiers PXD020156.

Supplementary Dataset 1 information

List of the bait-prey interactions identified in the TIM3 interactome of $CD4^{+}$ T cells isolated from $TIM3^{OST}$ mice. Each line shows an interaction between a bait and a prey and includes the corresponding p-values, enrichments and stoichiometries before stimulation and at each stimulation time point.

Supplementary references

1 Junttila, M. R., Saarinen, S., Schmidt, T., Kast, J. & Westermarck, J. Single-step Strep-tag purification for the isolation and identification of protein complexes from mammalian cells. *Proteomics* **5**, 1199-1203 (2005).

2 Roncagalli, R. *et al.* Quantitative proteomics analysis of signalosome dynamics in primary T cells identifies the surface receptor CD6 as a Lat adaptor-independent TCR signaling hub. *Nat Immunol* **15**, 384-392, doi:10.1038/ni.2843 (2014).

3 Soriano, P. The PDGF alpha receptor is required for neural crest cell development and for normal patterning of the somites. *Development* **124**, 2691-2700 (1997).

4 Pettitt, S. J. *et al.* Agouti C57BL/6N embryonic stem cells for mouse genetic resources. *Nat Methods* **6**, 493-495, doi:10.1038/nmeth.1342 (2009).

5 Voisinne, G. *et al.* Quantitative interactomics in primary T cells unveils TCR signal diversification extent and dynamics. *Nat Immunol* **20**, 1530-1541, doi:10.1038/s41590-019-0489-8 (2019).

6 Wolf, Y., Anderson, A. C. & Kuchroo, V. K. TIM3 comes of age as an inhibitory receptor. *Nat Rev Immunol* **20**, 173-185, doi:10.1038/s41577-019-0224-6 (2020).

SUPPLEMENTARY FIGURES

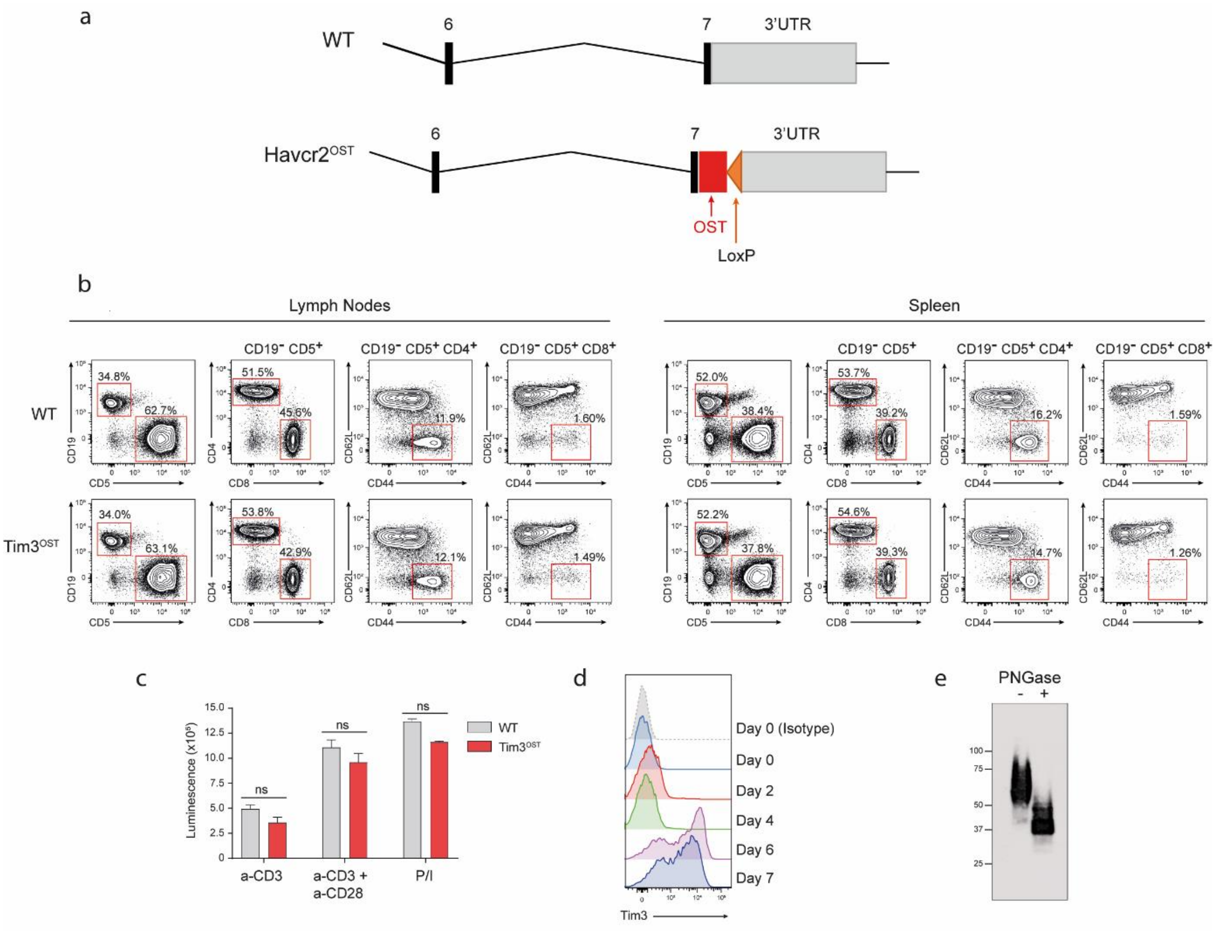

Figure S1. TIM3^{OST} mice contain normal T cells that show a normal TIM3 expression. **a** Structure of the 3' end of the WT *Havcr2* allele and of the *Havcr2*^{OST} allele. Exons correspond to transcript *Havcr2-201* (ENSMUST00000020668.14) are shown as filled black boxes and numbered. The Twin-Strep-tag coding sequence (OST, red) was inserted at the 3' end of exon 7 to generate the *Havcr2*^{OST} allele. The loxP site retained in the *Havcr2*^{OST} allele after excision of the ACN cassette is shown in orange. 3'UTR: 3' untranslated region. **b** The spleen and lymph nodes of WT and TIM3^{OST} mice were analyzed by flow cytometry for the expression of CD5 and CD19. CD19^{-}CD5^{+} T cells were analyzed for CD4 and CD8 expression, and the frequency of CD62L^{-}CD44^{+} and CD62L^{+}CD44^{-} T cells present among CD4^{+} and CD8^{+} T cells determined. The spleen and lymph nodes of TIM3^{OST} mice contained CD4^{+} and CD8^{+} T cells

in numbers comparable to that of WT mice and showed normal ratio of $CD62L^{-}CD44^{+}$ and $CD62L^{+}CD44^{-}$ T cells. Numbers indicate the percentage of specified cells. Data are representative of at least three experiments with three mice per group. **c** ATP content of purified $CD4^{+}$ T cells from lymph nodes of WT and $TIM3^{OST}$ mice activated for 48 h with plate-bound anti-CD3 antibody in absence (a-CD3), or presence (a-CD3 + a-CD28) of soluble anti-CD28 antibody, or with PMA and ionomycin (PI). ATP content was assessed by luminescence as a measure of the extent of cell proliferation. Data are representative of at least three experiments with three mice per group (mean and SEM are shown; ns, non-significant). **d** Purified $CD4^{+}$ T cells from WT mice were left unstimulated (day 0) or expanded and stimulated according to the workflow shown in figure 1b. $CD4^{+}$ T cells were analyzed by flow cytometry for TIM3 at the start of the culture (day 0), and after 2, 4, 6, and 7 days of culture. Grey shaded curves, isotype-matched control antibody (negative control). Data are representative of at least two experiments with three mice per group. **e** Purified T cells from $TIM3^{OST}$ mice were activated and expanded for 7 days as specified in figure 1b to induce optimal TIM3 expression. Cells were then lysed and total lysates subjected to affinity purification on Strep-Tactin Sepharose beads, followed by elution of proteins with D-biotin. Eluted proteins were incubated for 1 h at 37°C in presence (+) or absence (-) of PNGase F. Proteins were then analyzed by immunoblot with anti-TIM3 antibodies. Left margin: molecular size in kilodaltons (kDa). Data are representative of at least two independent experiments.

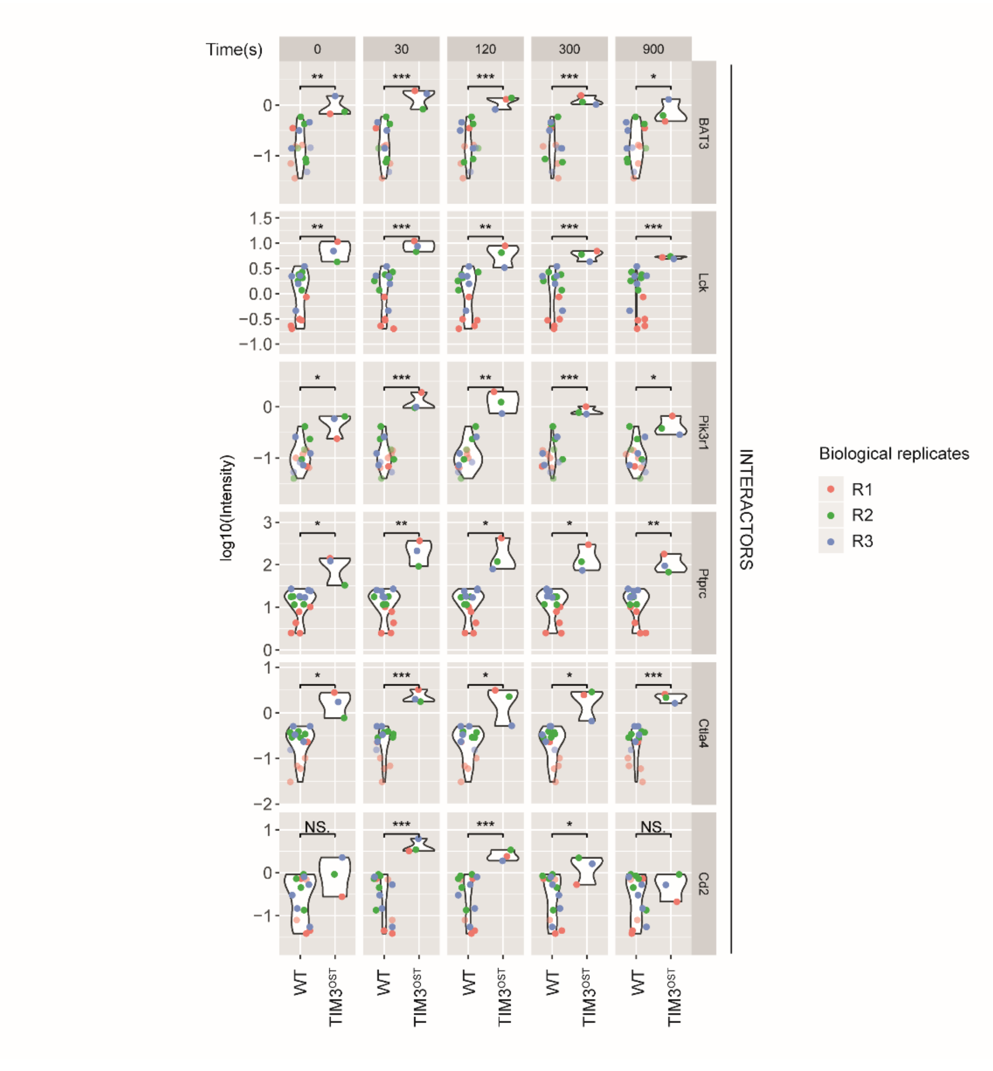

Figure S2. BAT3, LCK, PI3K p85α, CD45, CTLA-4 and CD2 are enriched in affinity purifications conducted in CD4$^+$ T cells from TIM3^{OST} mice as compared to control CD4$^+$ T cells isolated from WT mice. The abundances of the TIM3 interactors specified on the right were estimated for each time points and biological replicates (R1, R2 and R3). Normalized intensities (see Materials and Methods) from WT and TIM3^{OST} cells were compared using a two-sided Welch t-test (symbols used according to the t-test P-value: N.S., $P > 0.05$; $*P \leq 0.05$; $**P \leq 0.01$; $***P \leq 0.001$). Imputed missing values are represented with lighter shaded dots.

It has been proposed that in its ligand-unbound form, TIM3 interacts with BAT3 and promotes T cell activation via LCK recruitment[6]. Upon ligand engagement, Tyr256 and Tyr263 in the cytosolic tail of TIM3 are phosphorylated leading to BAT3 release and the recruitment of novel

intracytoplasmic effectors endowed with negative functions. As shown in the present figure, upon pervanadate treatment BAT3 and LCK remained associated to TIM3, suggesting that if a majority of TIM-3 molecules is phosphorylated, LCK and BAT3 association to TIM3 persists despite TIM3 phosphorylation.
